# Supplementary material for: Modeling glioblastoma heterogeneity as a dynamic network of cell states
Source: Mol Syst Biol. 2021 Sep 16;17(9):e10105. doi: 10.15252/msb.202010105 (PMC8444284; doi:10.15252/msb.202010105)
Supplement: Supplementary file 6 — Source Data for Figure 5 [file MSB-17-e10105-s004.zip › Figure5A_sourcedata/GSEA_3071/hallmarks_stateI.GseaPreranked.1623742531165/gsea_report_for_na_neg_1623742531165.html]

Report for na\_neg 1623742531165 [GSEA]

| GS  follow link to MSigDB | GS DETAILS | SIZE | ES | NES | NOM p-val | FDR q-val | FWER p-val | RANK AT MAX | LEADING EDGE || 1 | HALLMARK\_E2F\_TARGETS | Details ... | 138 | -0.53 | -2.93 | 0.000 | 0.000 | 0.000 | 1197 | tags=75%, list=34%, signal=110% |
| 2 | HALLMARK\_G2M\_CHECKPOINT | Details ... | 121 | -0.49 | -2.66 | 0.000 | 0.000 | 0.000 | 1346 | tags=78%, list=38%, signal=122% |
| 3 | HALLMARK\_MYC\_TARGETS\_V1 | Details ... | 88 | -0.45 | -2.27 | 0.000 | 0.000 | 0.002 | 1529 | tags=81%, list=44%, signal=139% |
| 4 | HALLMARK\_DNA\_REPAIR | Details ... | 46 | -0.50 | -2.24 | 0.000 | 0.000 | 0.003 | 1184 | tags=74%, list=34%, signal=110% |
| 5 | HALLMARK\_SPERMATOGENESIS | Details ... | 31 | -0.45 | -1.75 | 0.023 | 0.029 | 0.243 | 1097 | tags=61%, list=31%, signal=88% |
| 6 | HALLMARK\_KRAS\_SIGNALING\_DN | Details ... | 24 | -0.44 | -1.61 | 0.042 | 0.062 | 0.516 | 520 | tags=38%, list=15%, signal=44% |
| 7 | HALLMARK\_MYC\_TARGETS\_V2 | Details ... | 22 | -0.44 | -1.58 | 0.061 | 0.067 | 0.603 | 1346 | tags=73%, list=38%, signal=117% |
| 8 | HALLMARK\_ESTROGEN\_RESPONSE\_LATE | Details ... | 77 | -0.27 | -1.34 | 0.100 | 0.234 | 0.974 | 532 | tags=25%, list=15%, signal=28% |
| 9 | HALLMARK\_OXIDATIVE\_PHOSPHORYLATION | Details ... | 52 | -0.28 | -1.26 | 0.189 | 0.315 | 0.997 | 1677 | tags=67%, list=48%, signal=127% |
| 10 | HALLMARK\_KRAS\_SIGNALING\_UP | Details ... | 63 | -0.23 | -1.11 | 0.296 | 0.515 | 1.000 | 379 | tags=22%, list=11%, signal=24% |
| 11 | HALLMARK\_HEDGEHOG\_SIGNALING | Details ... | 25 | -0.27 | -1.00 | 0.449 | 0.676 | 1.000 | 147 | tags=12%, list=4%, signal=12% |
| 12 | HALLMARK\_UV\_RESPONSE\_UP | Details ... | 59 | -0.21 | -1.00 | 0.477 | 0.620 | 1.000 | 650 | tags=24%, list=19%, signal=29% |
| 13 | HALLMARK\_PEROXISOME | Details ... | 20 | -0.27 | -0.97 | 0.484 | 0.617 | 1.000 | 788 | tags=30%, list=22%, signal=38% |
| 14 | HALLMARK\_MITOTIC\_SPINDLE | Details ... | 87 | -0.19 | -0.97 | 0.506 | 0.579 | 1.000 | 1112 | tags=44%, list=32%, signal=62% |
| 15 | HALLMARK\_FATTY\_ACID\_METABOLISM | Details ... | 32 | -0.22 | -0.87 | 0.627 | 0.697 | 1.000 | 329 | tags=13%, list=9%, signal=14% |
| 16 | HALLMARK\_APICAL\_SURFACE | Details ... | 15 | -0.22 | -0.69 | 0.834 | 0.876 | 1.000 | 743 | tags=33%, list=21%, signal=42% |
Table: Gene sets enriched in phenotype **na **[plain text format]****
